# Epidemiological, Mitigation and Economic Impact of Zoonoses

**DOI:** 10.3390/ijerph182111704

**Published:** 2021-11-08

**Authors:** Beate Conrady

**Affiliations:** 1 Department of Veterinary and Animal Sciences, Faculty of Health and Medical Sciences, University of Copenhagen, 1870 Copenhagen, Denmark; bcon@sund.ku.dk; 2 Complexity Science Hub Vienna, 1080 Vienna, Austria

There is increasing evidence on the negative impacts of animal diseases on global productivity [1,2,3,4,5,6]. In addition to production losses, animal diseases cause great expense for the implementation of mitigation activities [7,8,9], as well as representing risks to human health associated with zoonoses (i.e., “any infection that is naturally transmissible from vertebrate animals to humans” [10]) [10,11,12,13]. Several previously unknown human infectious diseases have emerged from animals. In total, more than 200 zoonotic diseases have been reported worldwide [10,14]. In general, pathogen transmission can occur via different pathways, such as direct and/or indirect, vector and/or vehicle (e.g., food, air) contact [15,16]. The collection and analysis of epidemiological data regarding zoonotic diseases, as well as their respective intervention measures (such as control and/or eradication programs), is essential to detect infections and to interrupt infection cycles. Much still needs to be learned about the occurrence and spread of zoonoses pathogens, including their economic impact. 

The aim of this special issue is to improve the knowledge about the epidemiological, mitigation and economic impacts of zoonoses and beyond. To achieve this goal, seven papers (i.e., five research articles, one communication article and one review study) have been published in this special issue. The studies can be grouped into the following broad research areas: four studies focused on foodborne illness; one study analyzed vector-borne diseases; one study covered snakebites; and another study performed a literature review regarding the impact of coronavirus on society and culture. 

One of the most frequently reported zoonoses in the European Union is Salmonellosis [17]. Foodborne salmonellosis can cause (bloody) diarrhea and fever in humans. There are different transmission pathways for salmonellosis, such as domestic animals and contaminated eggs, meat and milk [17,18], as well as inconsistent cooking practices. Vajda and colleagues (2021) estimated the impact of foodborne salmonellosis on consumer well-being in Hungary by using the willingness-to-pay (WTP) method, i.e., how much money consumers would be willing to pay to avoid a Salmonella infection [18]. The presented study focused on nontyphoidal Salmonella infections. The authors used data from a quantitative consumer survey from 2017 (sample size: n = 1001) and compared the number of cases to reported cases in national statistics in order to estimate the occurrence of salmonellosis. Additionally, the survey data from 2019 (n = 1001) were used to calculate WTP. In this context, the impact of 13 sociodemographic characteristics (e.g., age, sex, whether children under the age of 15 were in the household and level of income) on WPT was investigated. The study results indicated that the occurrence of foodborne salmonellosis cases was 18 times higher than that officially registered in the national statistics. The average consumer WTP to avoid Salmonella infection was EUR 86.3 in Hungary. The study shows that there is a statistically significant difference between age groups, geographical locations, whether children under the age of 15 were in the household, level of education, and level of income and WTP. 

In total, the consumer’s well-being loss was estimated to be EUR 7.87 million per year in Hungary. The authors of this study concluded that, considering other foodborne pathogens also cause well-being loss for society, the implementation of prevention measures is essential.

Another study in this special issue investigated the epidemiological characteristics and spatiotemporal trends of human brucellosis (HB) in China from 1950 to 2018 [19]. The aims of this study were to predict HB incidences for the years 2019 and 2020, to describe the distribution of HB and to analyze the relationship between the spatial distribution of HB cases and gross domestic product (GDP) data in China. For this purpose, the authors used three longitudinal monitoring datasets covering: (i) the number and incidence of brucellosis cases by region for the period 1950–2013; (ii) the number and incidence of brucellosis cases stratified by age group and geographical regions for the period 2014–2018; and (iii) GDP data for the same period. The authors used different statistical approaches, such as the Holt–Winter exponential smoothing method, to forecast short-term HB incidences in the years 2019 and 2020 based on data from 2005 to 2018, or Moran’s I index to investigate whether HB case distribution patterns were clustered. The study results predicted declines in the HB incidences in 2019 and 2020. Comparing the number of reported HB cases with the prediction showed a model prediction accuracy of 80%. The relationship between the occurrence of HB and areas with high per capital GDP was negative from 2004 to 2018, i.e., there were no correlations between HB cases and GDP. Furthermore, the study shows that spatial autocorrelation and spatial clustering of HB incidences (2004–2018) existed in different regions in China. The spatial clustering varied by different provinces and was illustrated in different maps. Additionally, the authors observed an opposite trend in the spatial development of the incidence of HB. Although the incidence rate decreased in northern China, an increasing number of affected people with brucellosis have been observed in southern China since 2015. The authors highlighted that HB is highly dependent on the epidemic situation in animals and implemented intervention measures, which might explain the different distribution in HB cases between the north and south of China. 

Another research study dealt with Lyme disease, which represents one of the most important vector-borne diseases globally [20]. A steady increase in affected humans has been observed in the United States and no human vaccines are available; therefore, the motivation for the study was to improve the knowledge about the risk factors for Lyme disease infections [20,21,22]. In this context, Dong and colleagues (2021) compared the climate and landscape risk factors for Lyme disease in the Upper Midwest and Northeastern regions of the United States, where Lyme disease is endemic and transmitted by *Ixodes scapularis* (vector: deer ticks). The study analyzed the relationship between the annual number of human Lyme disease cases for each county from 2012 to 2016, and the predictor variables of climate, seven national land cover classes and the distance to the origin areas of Lyme disease. For this purpose, the authors used a generalized linear mixed model with negative binomial regression [22]. The model results indicated that in both analyzed regions the landscape factors (related to developed areas and forest) had similar effects on the occurrence of Lyme disease. In contrast to Northeast, the authors identified in the Upper Midwest, a relationship between the occurrence of Lyme disease and high precipitation as well as low temperatures. In general, the seasonal mean maximum temperature did explain the spatial patterns of Lyme cases in the model better compared to seasonal mean temperature. 

The study by Menconi et al., 2021, provides the first report of *Clinostomum complanatum* (Trematoda: Digenea) in European Perch *(Perca fluviatilis)* detected in an Italian subalpine lake in Lombardy [23]. *Clinostomum complanatum* is a fish-borne zoonotic parasite which causes Halzoun syndrome in humans after raw freshwater fish consumption. The study results showed that 21 (18.75%) out of 112 European Perch tested positive for encysted metacercariae in fillets of Perca fluviatilis. Biometric characteristics, i.e., total length and total weight, as well as sex and the presence of metacercariae, did not exhibit a correlation. The authors recommended further investigations of Perca fluviatilis as a source of human infection in other small lakes in Italy to improve the epidemiological data available for veterinary public health authorities regarding the reservoir for zoonotic parasites. 

In total, 2.7 million people every year are bitten by snakes worldwide [24]. The purpose of the study by Schneider and colleagues was to identify the racial group most frequently exposed to snakebites in Brazil. In this context, the authors investigated the association between the outcomes after a snakebite and seven covariables (i.e., race, location of the bite on the body, age, sex, received antivenom against the bites, case classification, time between accident and health care) by applying a binomial logistic regression model [24]. For their study, the authors used the Brazilian surveillance system which includes notifications of snakebites. In total, more than 58% of the snakebites occurred in rural areas in Brazil in 2017. The descriptive analysis showed that males were more often affected compared to females, and the average age was 36 years. More than half of all bites occurred in lower limbs, i.e., the feet, and were categorized as mild bites, and more than 79% bitten humans received antivenom. The fatality rate was 3.5 times higher in the indigenous group compared to the white population group, and the risk of being bitten was six times higher compared to the white population. Nonetheless, in the final multivariate model, only an association between the bad outcomes from snakebites (i.e., deaths and/or local complication and/or systemic complication) and the number of hours between accident and health care received and the severity of the case was identified. No association was detected with the other investigated covariables such as race. 

The study by Pavez-Muñoz et al., 2021, investigated risk factors for positivity to Shiga toxin-producing *Escherichia coli* (STEC) and *Salmonella enterica* in backyard production system (BPS) animals from the Metropolitana region in Chile [25]. An epidemiological survey regarding the general handling characteristics (e.g., consumption and/or sale of animal products), biosecurity characteristics (e.g., contact between visitors and BPS animals), and relationship with government agricultural entities (e.g., official veterinary service visits) was performed, and fecal samples were collected to detect both pathogens in BPS. Risk factors for STEC, *S. enterica* and for both pathogens were determined by using three multivariable logistic regression models. In this context, the Gini–Simpson index was calculated to consider the diversity of species present in BPS as a potential risk factor in the regression model as well. In total, 11.7% and 4.7% of all BPS (n = 85) were positive for STEC and *S. enterica*. The regression model results showed that: (i) the Gini–Simpson index and neighboring intensive poultry or swine production systems were statistically positively associated with STEC, i.e., an increased the risk of positive STEC; (ii) the exchange of embryonated eggs and the presence of debeaked chickens was significantly associated with *S. enterica* and an increased risk for *S. enterica*; (iii) the Gini–Simpson index and whether the BPS was a user of specific programs such as social development programs (Programa de Desarrollo Social) increased the risk of testing positive to both pathogens, whereas the type of confinement under which the animals are kept decreased the risk in cases where the animals were kept under a mixed confinement system. Based on the third regression model results, the authors concluded that there is a greater risk for positive pathogen occurrence if a BPS has more species. Another study performed a literature review regarding the impact of coronavirus on society and culture, which completes the special issue presented here [26]. The 22 included articles in the review can be broadly classified into three main topics: governance, knowledge and communication, as well as social aspects. The study highlighted the socio-cultural challenges associated with the COVID-19 pandemic. In conclusion, the present special issue has gathered seven research studies that provide new insights into the epidemiological, mitigation and economic impact of zoonoses. 
